# Semen Quality, Testicular Cell Apoptosis, and Transcriptome Analysis Following Mild Scrotal Heat Stress in Wugu–Hu Crossbred and Hu Rams

**DOI:** 10.3390/ani15050724

**Published:** 2025-03-03

**Authors:** Shikun Chen, Mingxu Jiang, Yanyun Wang, Qingjie Pan, Henry Annandale, Peter Charles Irons, Huansheng Dong

**Affiliations:** 1 College of Animal Science and Technology, Qingdao Agricultural University, Qingdao 266109, China; 34095258@student.murdoch.edu.au (S.C.); 17860824172@163.com (M.J.); wyy0408@outlook.com (Y.W.); qjpan@126.com (Q.P.); 2 College of Environmental and Life Sciences, Murdoch University, Murdoch, WA 6150, Australia; henry.annandale@murdoch.edu.au

**Keywords:** ram, sperm, spermatogenesis, heat stress, transcriptome, apoptosis

## Abstract

Hu and Wugu × Hu rams underwent scrotal insulation to simulate mild heat stress, resulting in a 3.0 ± 0.1 °C increase in scrotal surface temperature. Semen samples were collected every five days from day 11 to 56, and testis samples immediately after insulation. Both breeds experienced similar semen quality reductions and recovery trends, including reduced motility, concentration and the percentage of morphologically normal, but on days 41 and 46, Wugu–Hu rams exhibited significantly lower sperm motility than Hu rams (*p* < 0.05). Wugu–Hu rams demonstrate more transcriptomic changes. Further GO analysis revealed enrichment in spermatogenesis-related processes, while KEGG analysis identified Oocyte meiosis and cell cycle pathways, with a downregulation of key genes (CDK1, CDK2, CDC20, and PLK1) indicating impaired meiosis in Wugu–Hu rams. In contrast, Hu rams showed minimal transcriptional changes, contrary to the transcriptomic results. The significantly increased apoptosis rate of Wugu–Hu sheep testicular cells (*p* < 0.05) suggests compensatory or post-transcriptional mechanisms mitigating functional impacts caused by transcriptomic changes. The conclusion is that mild scrotal heat stress affects sperm quality and testicular gene expression. Wugu–Hu rams demonstrate greater transcriptomic sensitivity, but this does not show significant differences in semen quality recovery due to the compensatory mechanism of cell apoptosis.

## Simple Summary

This study explored how mild heat stress affects the semen quality and genetic activity of two breeds of rams: Wugu–Hu crossbred rams and Hu purebred rams. By placing scrotal insulation on six rams of each breed, researchers simulated a mild temperature increase and measured the effects over 56 days. The scrotal temperature increased, and testicular size did not change, but it did lead to a temporary drop in semen quality for both breeds. However, the crossbred Wugu–Hu rams showed more pronounced changes at the genetic level, suggesting they might respond differently to high temperatures. Overall, the findings indicate that while both breeds recover similarly in terms of sperm quality, Wugu–Hu rams may be more sensitive to heat at the cellular level.

## 1. Introduction

The earth’s climate is warming rapidly, reaching over 1.5 degrees Celsius above preindustrial levels in 2024 [1]. Over the last 20 years, there has been an increase in heat wave occurrences along the eastern coast of China [2]. Based on the prediction and evaluation model for future warming of 0.5 degrees, more high-temperature weather events are expected to occur [2].

Rising ambient temperatures have profound implications for animals. When the environmental temperature rises above the ‘prescriptive’ zone for the species, the total heat load exceeds the range of ambient temperatures over which an animal can maintain a stable body temperature through sensible heat loss alone, and heat stress results [3,4]. Heat stress may affect the entire body or have more local effects [5].

Of particular concern with increases in temperature is the effect on male breeding potential. Mammalian testes are very sensitive to temperature changes outside their normal range of 2 to 8 °C below core body temperature [6]. Frequent episodes of extreme heat weather may overload the thermoregulatory capacity of the animal’s scrotum, leading to heat stress in the form of elevated testicular temperatures [7]. The pathogenesis of the observed deleterious effects of elevated temperatures on testicular function is the subject of active research, and long-held beliefs have recently been challenged by new findings [8].

Testicular heat stress may affect all periods of spermatogenesis, resulting in the decline of semen quality reflected in reduced sperm motility, sperm count, and the percentage of morphologically normal sperm [9,10,11]. It is well documented that naturally occurring hot weather significantly reduces sperm quality in rams by raising the temperature of the testicles [12,13,14,15]. Scrotal insulation is a commonly used experimental model for studying testicular temperature increases without altering environmental temperature [16]. This experimental method has been widely used to study testicular heat stress in farm animals [16,17,18]. With the recent advancements in sensor technology, continuous temperature monitoring and infrared sensor detection have become increasingly popular [19,20]. Molecular and ‘omics’ technologies also provide new possibilities for studying responses to insults.

The adverse effects of heat stress on ram reproduction vary by breed [21,22]. Crossbreeding is a useful way of utilizing breed differences in heat tolerance to influence the heat tolerance of the offspring [23]. Wugu sheep (also known as black bone sheep), originating from Lanping county in Yunnan, China, are notable for their genetic trait of black pigmentation of both the skin and fleece and the internal organs including the bones [24]. The breed originated from an area of high altitude (2600 m on average) and rugged terrain characterized by a more variable climate relative to the typical conditions in the central-eastern regions.

Wugu sheep display later sexual maturity, smaller litter size, and lower reproductive rates and are therefore less productive for commercial farming as the Hu sheep breed popular in East China. However, due to the unique color and meat quality, Wugu sheep meat is favored by local consumers and is often priced at more than twice the price of Hu sheep meat in the area. To maximize sheep farming profitability, some commercial farms select F1 rams from Wugu ram × Hu ewe crosses, characterized by distinct black traits (including black eyelids, anus, tongue, and coat), as sires for the next generation of breeding. In addition, the Wugu breed also exhibits characteristics associated with significant heat tolerance, including pronounced pigmentation and a coarser coat [25,26], which are potentially advantageous in breeding animals suited to warmer environments. Farmers have observed that hybrid generations of Wugu sheep exhibit better tolerance to heat stress when used for breeding. However, there is no scientific research to support this observation. Should this be confirmed, it would favor the expansion of the Wugu breed, especially in hot areas where heat stress, caused by high ambient temperatures, is a crucial limiting factor affecting the efficiency of animal husbandry.

Transcriptomics technologies are techniques used to study the sum of all RNA transcripts [27]. The transcriptional gene regulation of testicular cells plays a crucial role in many physiological processes [28,29]. Liu, et al. [30] used RNA sequencing to investigate the effects of heat stress on gene expression in testicular tissues of dairy goats, revealing the role of the PI3K/AKT signaling pathway in testicular heat stress. Despite extensive studies on the genetic and molecular mechanisms of stress-induced testicular cell damage in various animals, research on breed-specific transcriptional differences in sheep remains limited.

Therefore, this experiment aimed to determine the effects of short-term mild heat stress on the sperm quality and testicular cell apoptosis of rams and to identify differentially expressed RNAs involved in spermatogenesis following such heat stress. Furthermore, we used housed purebred Hu and crossbred Wugu–Hu rams to investigate the differential responses between the breeds. These results help determine the factors that affect the heat resistance of ram testes.

## 2. Materials and Methods

### 2.1. Animals Welfare and Experimental Design

All animal procedures were conducted according to the ethical guidelines for the care and use of animals for scientific purposes. The Animal Ethics Committee at Murdoch University reviewed and approved the study (Approval Code: OS3447/23, Approval Date: 12 September 2023).

For semen quality, the experimental animals were six purebred Hu sheep (‘H’) and six Hu × Wugu sheep hybrids (‘W’), totaling 12 animals. For transcriptome and apoptosis analyses, testicular samples were collected from three Hu rams following scrotal insulation and three from a control group, and scrotal insulation and control groups of Wugu–Hu sheep (*n* = 3 per group), totaling 12 rams. The groups were named Hu sheep scrotal insulation (H-SI), Hu sheep control (H-CTR), Wugu–Hu sheep scrotal insulation (W-SI) and Wugu–Hu control (W-CTR), respectively. Adult rams 1.5 to 2 years of age managed in purpose-built housing on a commercial farm were used. All experimental animals were provided with identical nutrition, unrestricted access to clean drinking water, and housed under uniform environmental conditions throughout the study.

**Figure 1:**
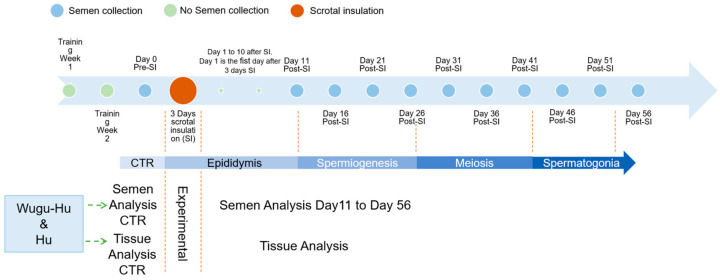
(Summary of the experimental procedures and phases of sperm production expected to be manifested in semen against time from scrotal insulation (SI). Semen collected on day 11 was presumed to be epididymal at SI, days 16, 21, and 26 reflect sperm in spermiogenesis at SI; days 31, 36, and 41 are the meiosis stage; and days 46 and 51 are presumed to be the spermatogonia stage.

The semen quality experiment was a clinical trial with four phases, namely a training phase, a pre-insulation phase, insulation phase, and post-insulation phase. Within sheep breed, semen quality data from the pre-insulation period were compared with data from the post-scrotal insulation period, thus serving as the point of reference (or control). Experimental procedures are summarized in Figure 1. Semen for evaluation was collected from each ram on Day 0 (CTR), followed by scrotal insulation for three days. Semen collection and evaluation were performed 10 times in each ram over 56 days at 5-day intervals beginning at 11 days after removal of the insulation bags.

### 2.2. Scrotal Insulation

The insulation bags were made of a breathable fabric and were close-fitting with a purse-string at the opening to retain the bags in position and prevent them from being easily removed. The insulation layer consisted of a double layer of cotton batting (Figure S1A). The scrotal insulation bag was designed with the aim of increasing the scrotal temperature by 2–4 degrees without affecting the rams’ health [31]. The thickness of the cotton filling material controlled the insulation effect. After measurement and adjustment, without compression, one side of the bag had a thickness of about 3 cm. The control group data for temperature was obtained using the same sensor device fixed in the same position with a single-layer bag with no insulation. This bag, made from a single piece of fabric, was open at the bottom to ensure breathability and contained no filling material. The experiment was performed in late September to early November, this being autumn when the weather in the region is generally mild.

A 3 cm × 3 cm × 0.5 cm sensor device (Bluetooth temperature sensor, JAALEE, Chengdu, China) was placed into an inner pocket of the insulation bags (Figure S1B) positioned at the middle on the posterior side of the scrotum.

These measured the temperature and humidity inside the insulation bag, closely opposed to the skin’s surface. The sensors recorded data every 20 min, continuously for 72 h, which was directly exported through the mobile application (JAALEE, Chengdu, China) via Bluetooth.

### 2.3. Temperature Records and Temperature–Humidity Index (THI)

The sheep farm is located at 35.7518° N, 119.2064° E, with four distinct seasons throughout the year. The experiment was conducted in autumn and lasted from late September to November. During the experimental period, no natural occurrences of extreme weather or high-temperature events were recorded.

The ambient temperature records were taken from the local weather station, and the relative humidity records were taken from the sensor for control group. The housing THI was calculated as follows: THI = (1.8 × Ambient temperature + 32) − (0.55 − 0.55 × Relative humidity × 0.01) × (1.8 × Ambient temperature − 26). The ram scrotal insulation THI was calculated as follows: THI = (1.8 × scrotal surface temperature + 32) − (0.55 − 0.55 × scrotal surface relative humidity × 0.01) × (1.8 × scrotal surface temperature − 26). Housing THI linked stress level: THI < 72 No stress; 72 ≤ THI < 78 Mild stress; 78 ≤ THI < 89 Moderate stress; THI ≥ 89 Severe stress [32].

### 2.4. Scrotal Circumference

The circumference of the scrotum was measured before and on days 1, 11, 31, and 51 after the scrotal insulation. The method was to grasp the neck of the scrotum with one hand and push the testes down, then position the tape measure snugly around the widest point of both testes. All measurements were taken by the same person to avoid inter-operator effects.

### 2.5. Semen Collection and Evaluation

Semen collection was performed by the artificial vagina (AV) method, and all collections were carried out by the same experienced collector during the same time period (9:00 am–10:00 am). All the rams used in this study were put through a two-week training period before scrotal insulation. The collection process is summarized as follows. Semen was collected using an AV consisting of a plastic or PVA tube (25 cm long, 7 cm in diameter) with a rubber inner liner. Warm water (39–42 °C) and air were added between the liner and tube to put moderate pressure on the penis. A collection vial was attached to one end. A restrained teaser ewe was introduced to the ram. Upon mounting, the operator positioned the AV in front of the ram’s penis and allowed the ram to mate into the AV to collect the ejaculate. Semen was collected and placed in 15 mL centrifuge tubes in a 37 °C water bath immediately. Laboratory evaluation started within 15 min of collection.

The semen was diluted 1 in 2000 using a 3% NaCl solution for the concentration (spermatozoa/mL) count, which was performed using a hemocytometer under 400× magnification.

Sperm total motility and progressive motility were performed with Mailang computer-aided sperm analysis (Model SQA-6100vet, Nanning, China) with image acquisition using a phase contrast microscope (CH3_DO 6M01434, Olympus, Japan).

Semen smears stained using a Diff-Quik staining kit (DA1210A, Leagene Biotechnology Co., Ltd., Beijing, China) were used to assess sperm morphology according to the methodology of Jin, et al. [33]. A phase contrast microscope (CKX53, Olympus, Tokyo, Japan) at 1000× magnification was used to evaluate the morphology of at least 200 spermatozoa. Sperm were categorized into the following morphological categories: head defects, midpiece defects, and tail defects [34].

### 2.6. Testicular Sample Collection for Transcriptome Analysis and Preparation of Paraffin Tissue Sections

Twelve rams destined for slaughter were selected as previously described, with three of each breed group subjected to scrotal insulation and three serving as controls. Testicular samples were collected immediately after the rams were slaughtered at commercial slaughterhouses. One sample group was obtained from the interior of the dissected testicle, including the seminiferous tubules and interstitial cells in the central region. Each sample weighed approximately 5 g and was snap-frozen in liquid nitrogen for preservation. Another sample group was fixed in a 50 mL centrifuge tube containing 4% paraformaldehyde (Solarbio, Beijing, China) for tissue paraffin sectioning

### 2.7. RNA Extraction and Library Preparation

Total RNA was extracted from testis samples using TRIzol reagent, and RNA purity was checked with a kaiaoK5500^®^ spectrophotometer (Kaiao, Guangzhou, China), while RNA integrity and concentration were assessed using the Agilent Bioanalyzer 2100 system (Agilent Technologies, Santa Clara, CA, USA). A total of 12 libraries were prepared using the NEBNext^®^ Ultra™ RNA Library Prep Kit (New England Biolabs, Ipswich, MA, USA), including 3 Hu ram heat stress libraries, 3 Hu ram control libraries, 3 Wugu–Hu ram heat stress libraries, and 3 Wugu–Hu ram control libraries. Each library was prepared with 2 μg of RNA, followed by mRNA purification, cDNA synthesis, fragmentation, tailing, adapter ligation, and PCR amplification. Libraries were quantified using Qubit^®^ 3.0 (Thermo Fisher Scientific, Waltham, MA, USA) and Agilent Bioanalyzer 2100, followed by cluster generation using the cBot system (Illumina, San Diego, CA, USA). Sequencing was performed on the Illumina platform with 150 bp paired end reads.

### 2.8. Quality Control and Gene Expression Quantification

Raw data underwent quality control using a Perl script to remove short reads, adapter contamination, and low-quality reads. Gene expressions were quantified using HTSeq to count the reads for each gene, and fragments per kilobase of transcript per million mapped reads (FPKM) was calculated to estimate expression levels. Differential expression analysis was performed using DESeq2, which uses a negative binomial distribution model and linear regression for gene expression estimation. Wald tests were used to calculate *p*-values, and the Benjamini–Hochberg method was applied for *p*-value correction.

### 2.9. Function Enrichment Analysis

The gene ontology (GO) enrichment analysis of differential genes was implemented by the R package clusterProfiler. For enrichment analysis with this package, the corrected *p*.adjust ≤ 0.05 by BH method was selected as the threshold and a GO term that satisfies this condition is defined as a GO term that is significantly enriched in differentially expressed genes. GO functional significance enrichment analysis can identify the main biological functions performed by differentially expressed genes.

Kyoto encyclopedia of genes and genomes (KEGG) is a comprehensive database that integrates genomic, chemical, and systemic functional information. Use ClusterProfiler to calculate the significantly enriched map pathways in the target gene.

Enriched GO and KEGG pathways were nominally statistically significant at the *p* < 0.05 level. The construction of protein–protein interaction (PPI) networks was also conducted by using the STRING database with the Cytoscape software v3.10.3 (Institute for Systems Biology, Seattle, WA, USA).

### 2.10. Real-Time Quantitative Polymerase Chain Reaction

Reverse transcribe RNA into cDNA using the SynScript III RT SuperMix kit (TSK-314, Beijing Tsingke Biotech Co., Ltd., Beijing, China). Subsequently, following the instructions of ArtiCanATMSYBR qPCR Mix kit (TSE501, Beijing Tsingke Biotech Co., Ltd., Beijing China), real-time quantitative polymerase chain reaction (RT-qPCR) was performed using SYBR Green I on the Bio Rad CFX Maestro system. The reaction mixture contains 10 μL of SYBR Green mixture, 8 μL of RNA free water, 0.5 μL of upstream and downstream primers, and 1 μL of cDNA. The amplification conditions include an initial denaturation step at 95 ° C for 1 min, followed by 10 s at 95 ° C and 20 s at 60 ° C for 40 cycles. The experiment includes 5 biological replicates. The primers are shown in Table S2.

### 2.11. TUNEL Assay for Detecting Apoptosis

Paraffin tissue sections were prepared as follows. After 12 h, the tissue fixed in paraformaldehyde was sequentially dehydrated in 30%, 50%, 75%, 90%, 95%, 100% (I), and 100% (II) alcohol (Fuyu, Tianjin, China), with each gradient lasting 30 min. The tissue was then placed in xylene (Fuyu, Tianjin, China) for 20 min before being immersed in paraffin with a melting point of 56 °C for 12 h. Finally, the tissue was embedded in paraffin for sectioning, and slices were prepared using a paraffin rotary slicer (SN02026, Leica Microsystems, Shanghai, China) to a thickness of 4 μm.

A TUNEL cell apoptosis detection kit (Cat.C1091, Beyotime, Shanghai, China) was used for cell apoptosis detection. Paraffin sections were deparaffinized in xylene (10 min × 2) and rehydrated through a graded ethanol series (at 100%, 90%, and 70%) followed by distilled water (2 min). Antigen retrieval was performed using 20 μg DNase-free proteinase K at 32 °C for 20 min, followed by three PBS washes (2 min each). Endogenous peroxidase activity was quenched with 3% H_2_O_2_ (20 min at room temperature), then sections were washed three times with PBS. Biotin labeling solution (50 μL) was applied and incubated at 37 °C in the dark for 60 min, followed by a PBS wash and treatment with 0.2 mL of stop solution (10 min, room temperature). After three PBS washes, 50 μL of Streptavidin-HRP solution was added for 30 min at room temperature. Sections were then incubated with 0.5 mL DAB substrate (15 min at room temperature) and counterstained with hematoxylin before observation. Count the number of TUNEL positive cells and the total number of cells per seminiferous tubule, presented as the percentage of positive cells.

### 2.12. Statistical Analysis

The results for each day of collection were summarized as the means for each breed of ram ± standard error with confidence intervals calculated at 99%. Statistical analyses were conducted using the software GraphPad Prism 10.0 (GraphPad Software, Inc., Boston, WA, USA). *t*-test was used to compare the difference between the sperm motility; differences between groups were considered significant when *p* < 0.05 and highly significant *p* < 0.01.

## 3. Results

### 3.1. Surface Temperature of the Scrotum

**Figure 2:**
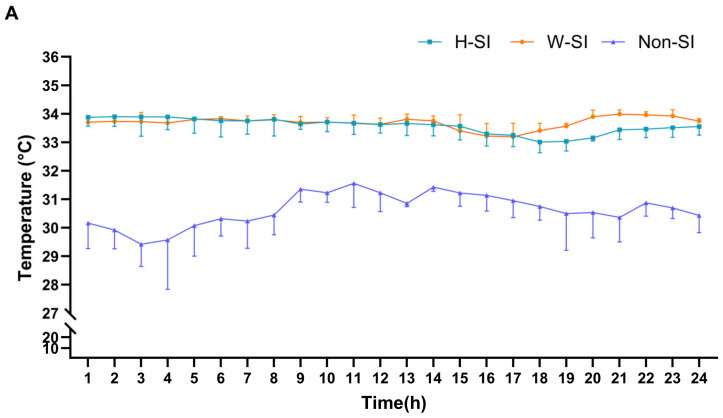

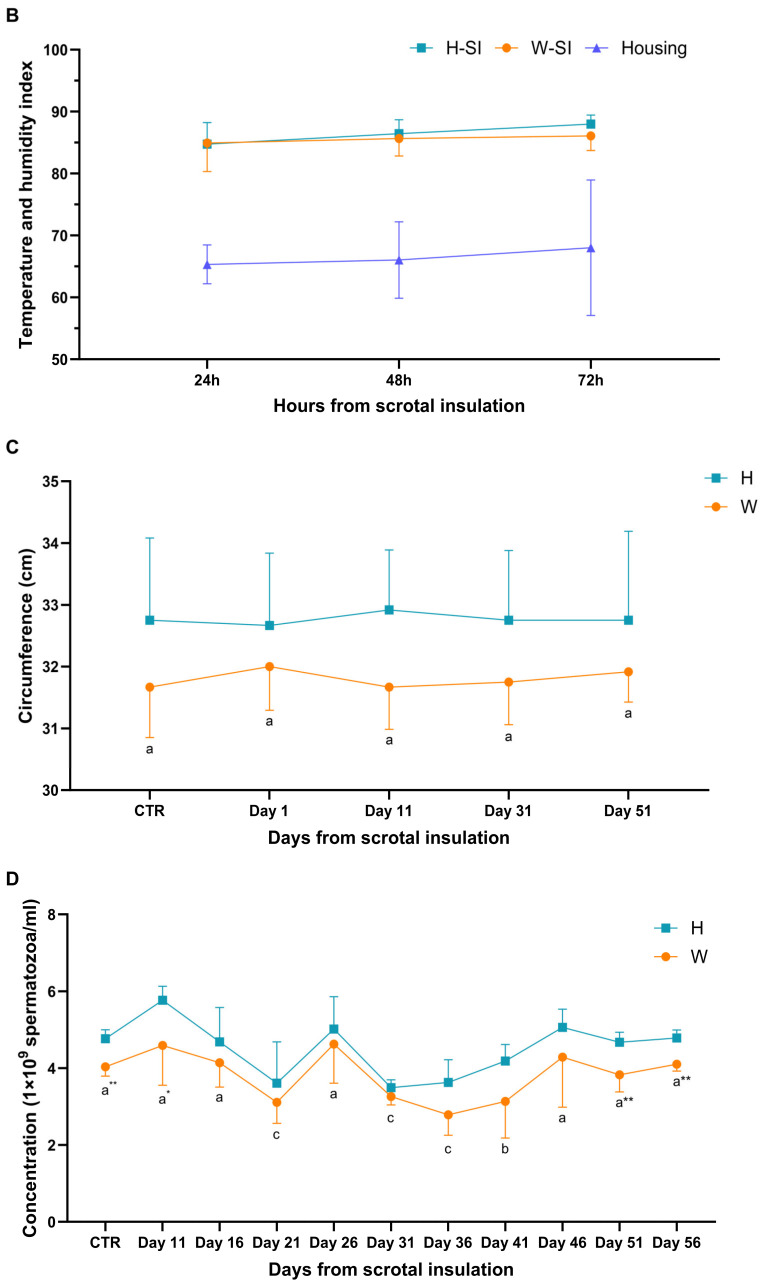
(**A**) Scrotal surface temperature. Each hourly data point is the mean of all measurements for all rams in the 3 recordings in each hour and the 3 days of recording. (**B**) Comparison between housing THI and scrotal insulation THI. (**C**) Scrotal circumference. (**D**) The semen concentration. CTR: pre-scrotal insulation; Day 11–56: post-scrotal insulation; H: Hu ram; W: Wugu–Hu ram; H-SI: Hu ram with scrotal insulation bag; W-SI: Wugu–Hu ram with scrotal insulation bag; non-SI: ram without scrotal insulation. a, b, c: Differing subscripts denote a significant difference within group compared to CTR, with a: no significant difference (*p* > 0.05); b: significant difference (*p* < 0.05); and c: highly significant difference (*p* < 0.01). * Significant difference between groups on the same day (*p* < 0.05); ** highly significant difference (*p* < 0.01). Lack of differing superscripts indicates *p* > 0.05.

The average surface temperature of the scrotum for Hu sheep scrotal insulation group (H-SI), Wugu–Hu sheep scrotal insulation group (W-SI), and control group (Non-SI) across the 72 h of insulation were 33.58 ± 0.94 °C, 33.69 ± 1.02 °C, and 30.63 ± 2.54 °C, respectively, with little diurnal variation in the insulation groups (Figure 2A). There was no difference (*p* = 0.10) between the scrotal temperatures of Hu and Wugu–Hu rams. The temperature of the two scrotal insulation groups was higher than that of the controls (*p* < 0.01).

### 3.2. Housing and Ram Scrotal Insulation THI

During the experiment, the average high temperature in the region of the farm where the experiment was conducted was 23 °C, and the average low temperature was 16 °C, with the maximum of 27 °C on day 29 and the minimum (13 °C) on day 25 [35].

During the scrotal insulation period, the average ambient THI inside the housing remained below 72, and there was no difference between the three days (Figure 2B). The THI inside the scrotal insulation was significantly higher than the housing THI on each day (*p* < 0.01).

### 3.3. Scrotal Circumference and Semen Concentration

Figure 2C illustrates the changes in scrotal circumference over time in two groups of sheep following scrotal insulation. Wugu–Hu rams (group W) scrotal circumference was not different to that of Hu rams (group H) (*p* > 0.05). The mean circumference of group W was 31.8 cm (99% CI: 31.49 to 32.11 cm). The mean circumference of group H was 32.77 cm (99% CI: 32.58 to 32.95 cm).

Figure 2D shows the variation in sperm concentration. Significant differences to the CTR were observed on Days 21, 31, and 36 (*p* < 0.01) and Day 41 (*p* < 0.05) in group W. Compared to pre-insulation values, significant differences were observed on Days 11, 21, 31 and 36 (*p* < 0.01) and Day 41 (*p* < 0.05) in group H. In comparison between the two groups, significant differences were observed on CTR (*p* < 0.01), Day 11 (*p* < 0.05), and Days 51 and 56 (*p* < 0.01). The mean concentration in post-insulation was 3.79 × 10^9^ count/mL (99% CI: 3.10 × 10^9^ to 4.47 × 10^9^ count/mL) for group W and 4.49 × 10^9^ count/mL (99% CI: 3.72 × 10^9^ to 5.25 × 10^9^ count/mL) for group H.

### 3.4. Sperm Motility

**Figure 3:**
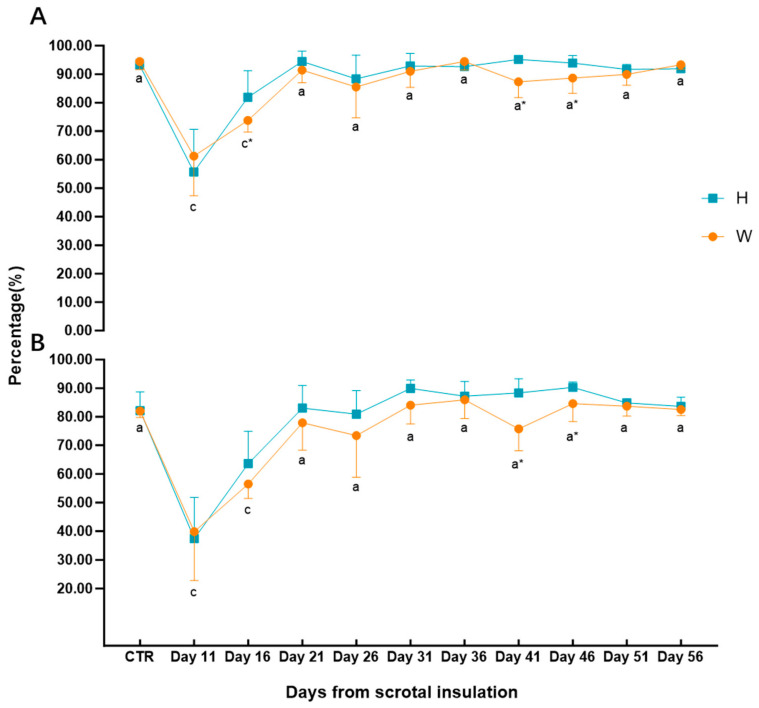
(**A**) The total sperm motility. (**B**) The progressive sperm motility. H: Hu ram; W: Wugu–Hu ram. a, c: Differing subscripts denote a significant difference compared to CTR. “a” stands for no significant difference (*p* > 0.05), and “c” stands for highly significant difference (*p* < 0.01). * Significant difference between groups on the same day (*p* < 0.05).

Compared to pre-insulation values (CTR), there was a significant post-insulation decrease in sperm total motility in Hu rams (group H) on Days 11 and 16 (*p* < 0.05), returning to pre-scrotal insulation levels on Day 21 and remaining stable (Figure 3A). Changes in Wugu–Hu (group W) and Hu rams (group H) were similar, with lower total sperm motility in Wugu–Hu rams on Days 16, 41, and 46, than Hu rams (*p* < 0.05) (Figure 3A). The mean total motility in post-insulation was 85.66% (99% CI: 75.02% to 92.30%) for group W and 87.87% (99% CI: 75.60% to 100.10%) for group H.

Compared to pre-insulation (CTR), there was a significant post-insulation decrease in progressive motility in Hu rams (group H) on Days 11 and 16 (*p* < 0.05), returning to pre-scrotal insulation levels on Day 21 and remaining stable (Figure 3B). Changes in Wugu–Hu (group W) and Hu rams (group H) were similar, with lower total sperm motility in Wugu–Hu rams on Days 41 and 46 than Hu rams (*p* < 0.05) (Figure 3B).

### 3.5. Sperm Morphology

**Figure 4:**
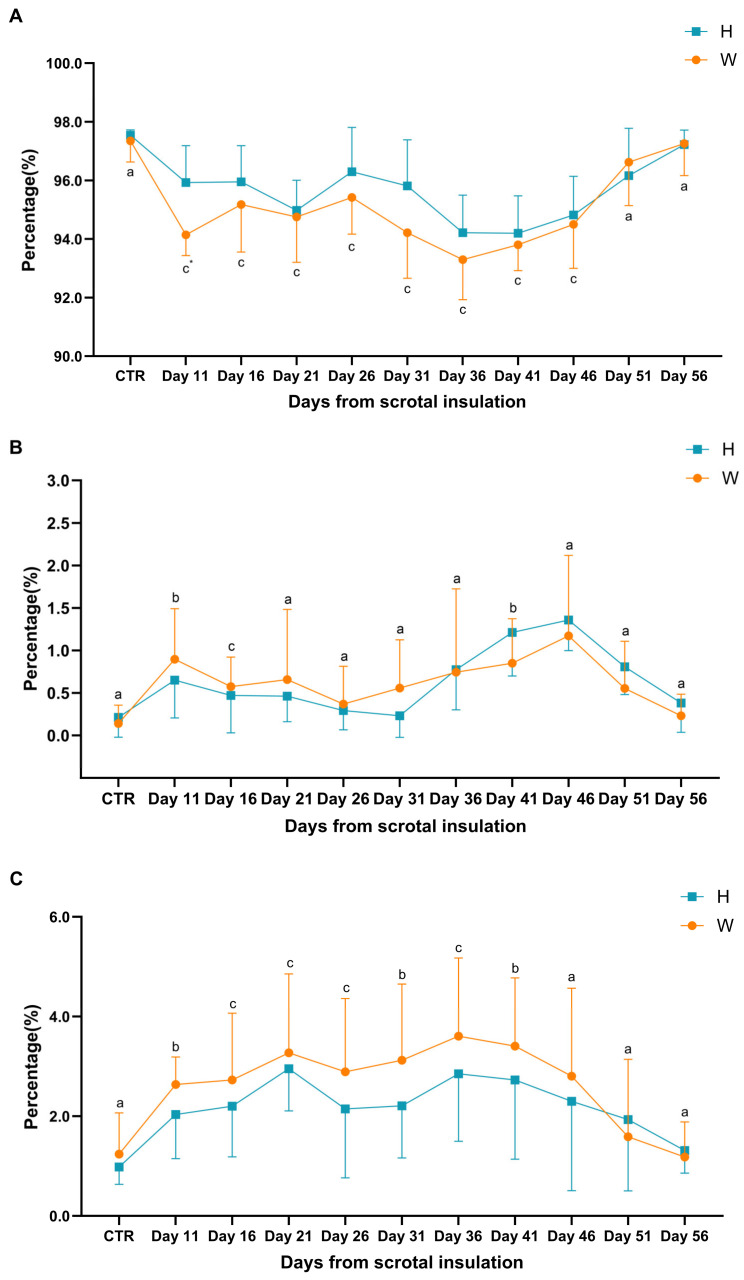

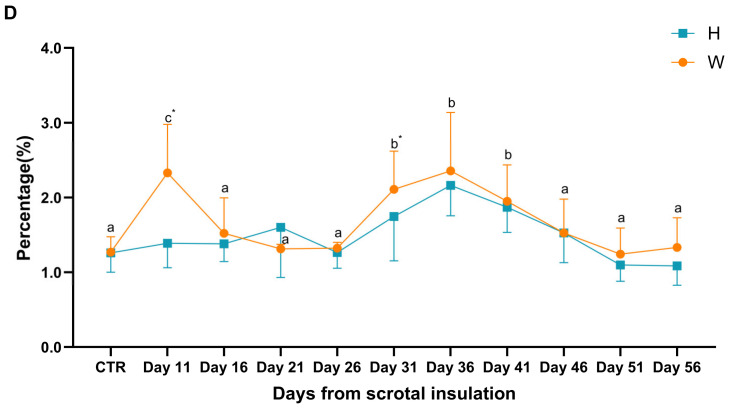
(**A**) Percentage morphologically normal sperm. (**B**) Percentages with head defects. (**C**) Midpiece defects. (**D**) Tail defects. a, b, c: Differing subscripts denote a significant difference compared to CTR. “a” stands for no significant difference (*p* > 0.05), “b” stands for significant difference (*p* < 0.05), and “c” stands for highly significant difference (*p* < 0.01). * Significant difference between groups on the same day (*p* < 0.05).

The percentage morphologically normal results are shown in Figure 4A. The W group showed highly significant differences from controls on Days 11, 16, 21, 26, 31, 36, 41, and 46 (*p* < 0.01), while the H group differed significantly on Days 11, 16, 21, 36, 41, and 46 (*p* < 0.01). A significant difference between the H and W groups was observed on Day 11 (*p* < 0.05).

Figure 4B shows the percentage of sperm showing head defects. The W group showed significant differences from controls on Days 11 (*p* < 0.05), 16 (*p* < 0.01), and 41 (*p* < 0.05). The H group showed significant differences on Days 36 (*p* < 0.05), 41 (*p* < 0.01), and 46 (*p* < 0.05). No significant differences were observed between the H and W groups.

Figure 4C shows the sperm midpiece defect rates. Compared to controls, the W group showed significant differences on Day 11 (*p* < 0.05), Days 16, 21 and 26 (*p* < 0.01), Day 31 (*p* < 0.05), Day 36 (*p* < 0.01) and Day 41 (*p* < 0.05). Compared to CTR group, the H group showed significant differences on Days 11 and 16 (*p* < 0.05), Day 21 (*p* < 0.01), and Days 31, 36 and 41 (*p* < 0.05). Comparing the H group to the W group on each collection day, no significant differences were seen (*p* > 0.05).

Figure 4D shows the sperm tail defect rates. Compared to controls, the W group showed significant differences on Day 11 (*p* <0.01), Days 31, 36 and 41 (*p* < 0.05). Compared to CTR group, the H group showed highly significant differences on Days 36 and 41 (*p* < 0.01). Comparing the groups, they both reached the maximum on Day 36 (2.36% and 2.16%) with no significant difference (*p* = 0.32). They show significant differences on Days 11 and 31 (*p* < 0.05).

### 3.6. RT-qPCR Validation

**Figure 5:**
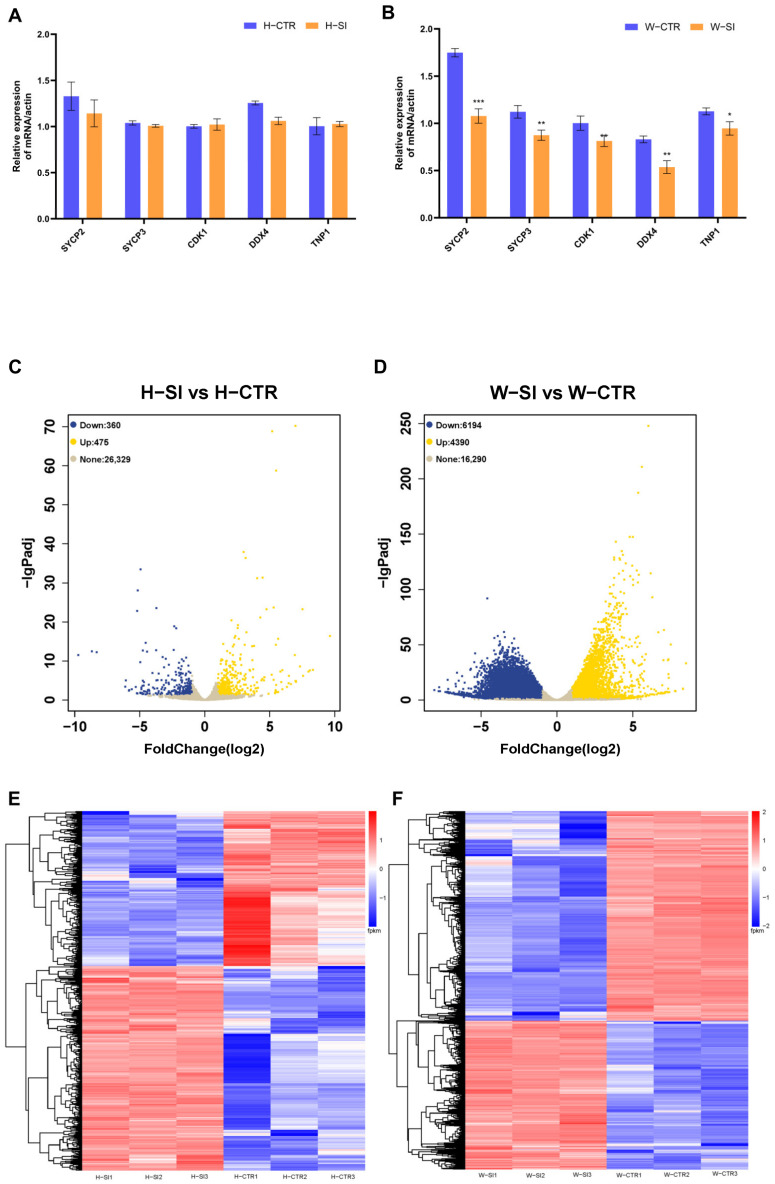
(**A**,**C**,**E**) RT-qPCR, volcano plot and heat map of DEGs in H-SI vs. H-CTR. (**B**,**D**,**F**) RT-qPCR, volcano plot and heat map of DEGs in W-SI vs. W-CTR. (**E**,**F**) Rows represent individual genes, and columns correspond to samples, with red indicating high gene expression and blue indicating low expression genes. The horizontal axis displays the clustering of the samples, and the vertical axis represents the clustering of the genes. * *p* < 0.05, ** *p* < 0.01, *** *p* < 0.001; Lack of differing superscripts indicates *p* > 0.05.

Five Differentially Expressed Genes (DEGs) were selected for validation through RT-qPCR analysis, namely SYCP2, SYCP3, CDK1, DDX4, and TNP1 (Figure 5A,B). The results were broadly consistent with those obtained from RNA sequencing (Table S3). These DEGs are associated with the key features selected for further discussion.

### 3.7. Transcriptome Characteristics of Ram Testis

The screening criteria for differentially expressed genes in this project are fold change ≥ 2, *p*-value ≤ 0.05, and padj ≤ 0.05. When comparing the H-SI and H-CTR groups, 835 differentially expressed genes were identified, with 475 upregulated and 360 downregulated (Figure 5C). In the comparison between the W-SI and W-CTR groups, 10,584 differentially expressed genes were identified, with 4390 upregulated and 6194 downregulated (Figure 5D).

Figure 5E,F show the differential gene clustering heatmaps of H-SI vs. H-CTR group and W-SI vs. W-CTR group, respectively. This analysis reveals distinct differential expression patterns in testicular tissue genes following mild scrotal heat stress while maintaining consistency among the replicates within each sample group. All DEG sets of the repeat groups are closely clustered, suggesting that rams of the same breed exhibit consistent gene expression patterns in this study. The clustering pattern indicates distinct transcriptomic responses in the two breeds. Wugu–Hu rams exhibited a broader spectrum of DEGs, as reflected by the greater number of upregulated and downregulated transcripts in Figure 5F compared to Figure 5E.

### 3.8. Function Analysis of DEGs

**Figure 6:**
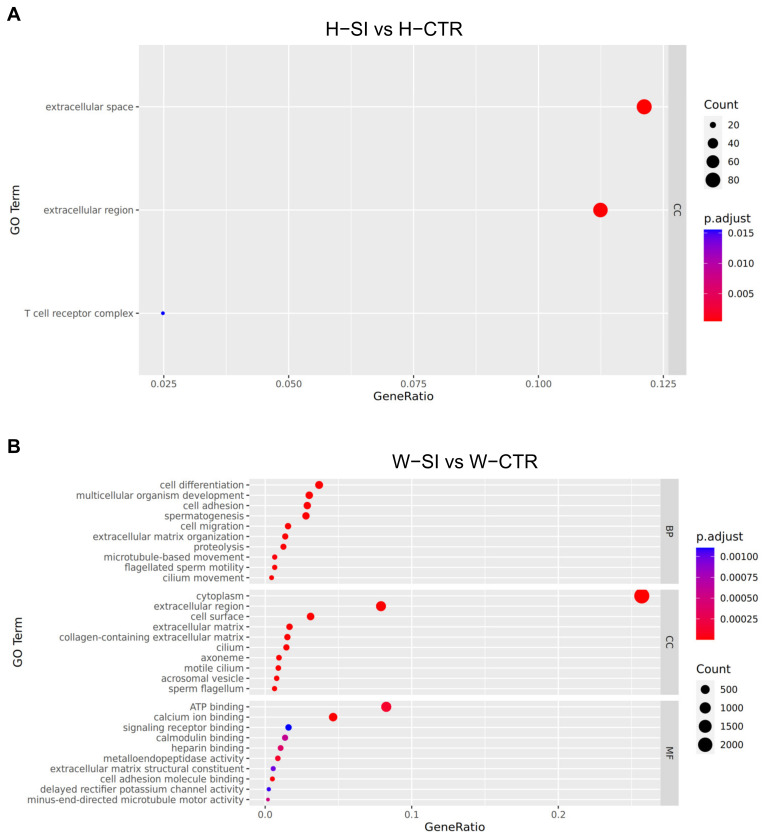

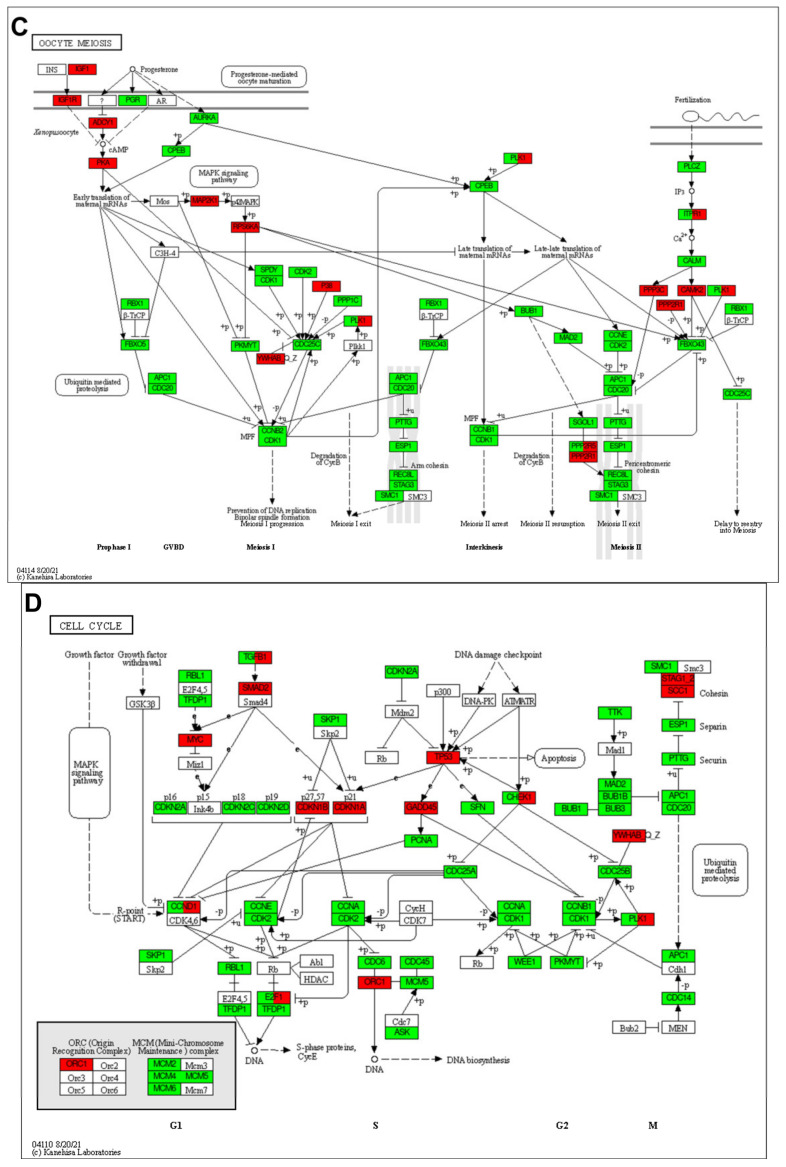
(**A**) GO enrichment analysis of H-SI vs. H-CTR. (**B**) GO enrichment analysis of W-SI vs. W-CTR. The vertical axis represents GO terms, while the horizontal axis indicates the proportion of genes enriched in each term relative to the total number of genes. The color denotes the adjusted *p*-value (*p*.adjust), with deeper red colors signifying higher significance. The size of the dots reflects the number of genes enriched in each GO term, with larger dots indicating a greater number of genes. (**C**,**D**) DEGs in the W-SI vs. W-CTR comparison are mapped onto KEGG pathways. The red-labeled genes are significantly upregulated (*p* < 0.05), while the green-labeled genes are significantly downregulated (*p* < 0.05). The white labeled genes are not differentially expressed (*p* > 0.05). Solid Arrows (→): Indicate direct activation or interaction between components. Dashed Arrows (⇢): Represent indirect interactions or regulatory relationships. Blocked Lines (⊥): Represent inhibition or suppression of gene/protein activity.

GO is divided into three ontologies: Molecular Function (MF), Cellular Component (CC), and Biological Process (BP). In this analysis, the top 10 most significant categories (or all categories if fewer than 10) were selected for visualization of the GO enrichment analysis results using a dotplot, as shown in Figure 6A (H-SI vs. H-CTR) and Figure 6B (W-SI vs. W-CTR).

KEGG pathway enrichment analysis was performed for the two comparison groups. In the Hu sheep group, no pathways were significantly enriched for DEGs, but multiple pathways were significantly enriched in the Wugu–Hu sheep group. Further analysis of the W-SI vs. W-CTR comparison using the KEGG pathway database identified two significantly enriched pathways related to spermatogenesis: Oocyte meiosis and cell cycle (Figure 6C,D). Seventy-five DEGs in the Oocyte meiosis pathway were significantly enriched (*p* = 0.0002), including 27 upregulated DEGs and 48 downregulated DEGs (Table S4). Seventy-two DEGs in the cell cycle pathway were significantly enriched (*p* = 0.015), comprising 21 upregulated DEGs and 51 downregulated DEGs (Table S4).

**Figure 7:**
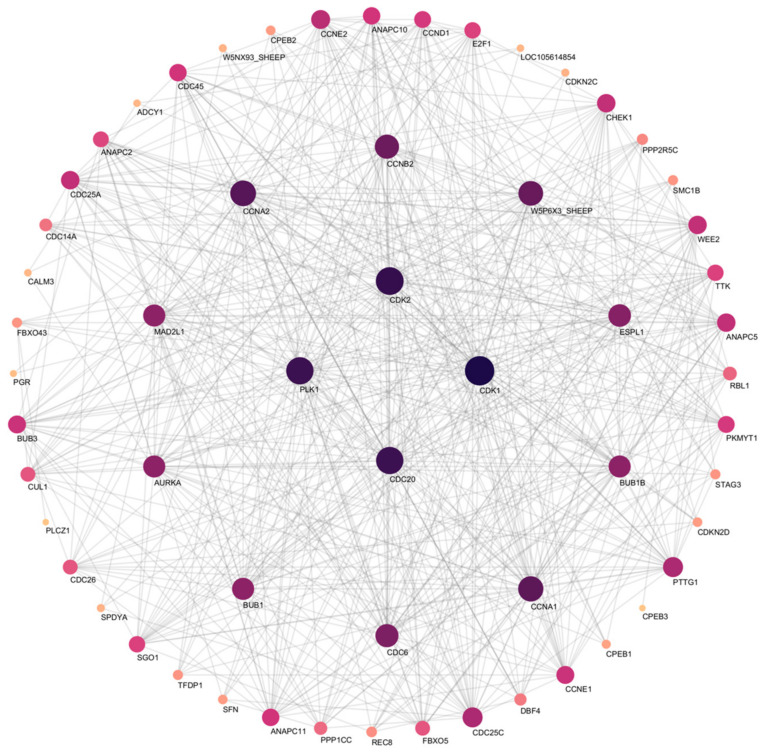
PPI network analysis. Node size and color intensity correspond to the degree of connectivity, with darker and larger nodes representing genes with higher interaction degrees.

Downregulated DEGs from both pathways were subjected to PPI network analysis. The network highlights the central roles of key genes, including CDK1, CDK2, CDC20, and PLK1, which are essential regulators of the cell cycle and spermatogenesis (Figure 7).

### 3.9. The Effect of Scrotal Heat Stress on Testicular Cell Apoptosis

**Figure 8:**
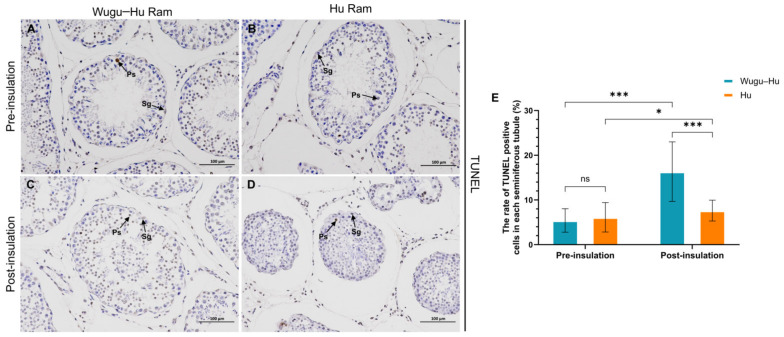
(**A**–**D**) TUNEL staining of testicular tissue slices. (**E**) The percentage of TUNEL positive cells in each seminiferous tubule. TUNEL positive cells are brown in color. Sg: spermatogonium; Ps: primary spermatocyte. Scale bars = 100 μm; magnification: 200×. * indicates *p* < 0.05; *** *p* < 0.001; ns *p* > 0.05.

A total of 50 seminiferous tubules were randomly selected from each sample group for TUNEL analysis of cell apoptosis. TUNEL positive cells are demonstrated in Figure 8 A–D. The proportion of TUNEL positive cells per seminiferous tubule was higher after scrotal insulation in both the Wugu–Hu and the Hu rams (Figure 8E), with *p* < 0.01 and *p* < 0.05, respectively (Figure 8E). The Wugu–Hu post-insulation shows a significantly higher rate of TUNEL positive cells compared with Hu ram post-insulation. The results indicate that mild scrotal heat stress increased testicular cell apoptosis of both sheep breeds at different levels.

## 4. Discussion

In this study, we were able to continuously monitor temperature and humidity on the scrotal surface during scrotal insulation, thereby confirming a continuous mild elevation of the scrotum despite the diurnal variations in the surroundings.

The average temperature measured in the middle of the caudal scrotal surface without insulation was 30.6 °C, which is consistent with previous studies [36]. With the insulation bags, the surface temperature of the scrotum increased by about 3 °C and was maintained steadily for three days until the scrotal insulation was removed. Compared with other studies, which applied scrotal temperature elevations of 2–4 °C for 32 to 68 h, the current experiment was calibrated to be at a lower level of scrotal heat stress [37]. An average ambient THI of less than 72 during the scrotal insulation period indicates that the environment would not have caused the rams to experience heat stress. Although the THI briefly reached 75.75 on the third day, it remained within the range of mild heat stress. In this study, the THI inside the scrotal insulation bag was calculated using the same formula. Although this value does not apply to the stress-level evaluation range for whole-animal exposure, it demonstrated that scrotal insulation caused a significant increase in local THI. The changes in humidity are shown in Figure S2.

Spermatogenesis is a 47–56 day process of primordial germ cells giving rise to spermatozoa through a series of sequential cell divisions and differentiation [38,39], with discrete insults to cells at each stage corresponding to effects in the ejaculated semen as indicated in Figure 1. When the temperature of the testicles increases, spermatogenic cells, germ cells, and sperm in the testes may be affected; therefore, semen quality will continue to be affected for some time, even after the heat stress has ended [40,41]. This is also consistent with the results of this study.

Both the Wugu–Hu and the Hu group showed similar responses in sperm progressive motility and total motility. The lowest average percentage of progressive and total motility was shown on the first collection (Day 11) in both groups. From this point, there was a gradual recovery trend, with the level returning to similar levels as the control group by Day 21 and subsequently maintaining a relatively stable state. Results from a similar experiment showed that the sperm’s lowest progressive motility was observed on the 21st day and recovered on the 35th day [31]. Although this timing differs, our results are similar in that the lowest viability in both experiments appeared in the first collection and recovered after 3 to 4 collections. Therefore, scrotal insulation had the greatest impact on the motility of sperm stored in the epididymis. Furthermore, this impact is transient when the insult is mild and brief as in this study. Because the sperm stored in the epididymis is not completely discharged in one ejaculation [42], the proportion of sperm with impaired motility decreases and the proportion of motile sperm increases as epididymal stores are replenished from the testes. The recovery time of sperm motility is therefore likely to be related to the number of semen collections and may be shorter than the 21 days seen in this experiment. However, this is only true for mild short-duration scrotum heat stress. When the scrotum temperature increased more than 4 °C [43], recovery of sperm motility took longer.

The semen concentration of both sheep breeds was significantly lower than the control (except Day 26) from Day 21 to Day 41 and recovered to the average level on Day 46. Such long-term decreases in concentration (<3 × 10^9^ sperm/mL) may lead to a drop in production efficiency, such as reduced fertilization rates [44,45]. We speculate that this long-term decreased concentration corresponds to heat stress caused by germ cell apoptosis. Heat stress can cause cell damage in the testes, leading to increased apoptosis of germ cells, and resulting in decreased sperm concentration [46,47]. Results of our TUNEL assays in testes tissue sections showed similar changes (Figure 8). In addition, heat stress can also reduce sperm cell proliferation due to impaired Sertoli cells function and contribute to a decrease in sperm concentration [48,49].

Although the percentage morphologically normal sperm (PMN) remained above 90% in both breeds, which is considered an ideal range, a reduction was seen, indicating that even this mild degree of scrotal heat stress affected testicular germ cells. It is noteworthy that sperm defects predominantly occur in the midpiece. Midpiece defects significantly increased before Day 46. One explanation for this change is attributed to the rise in temperature leading to testicular hypoxia, which indirectly increases reactive oxygen and negative influences in mitochondrial formation and function [6]. Another study suggested that increased deformity rates are due to the direct impact of temperature on sperm cells [50].

Transcriptome analysis provides more information for testicular response to mild heat stress. The comparison of the total number of DEGs after heat treatment demonstrates the different responses to mild heat stress between the two sheep breeds. After being subjected to mild heat stress, the Wugu–Hu rams showed more significant gene expression changes in the testes than the Hu rams. In GO analysis, the Hu-SI vs. Hu-CTR group only enriched three terms in CC ontology, and the W-SI vs. W-CTR group enriched over ten terms in each different ontology. Similarly, KEGG enriched pathways were only observed in the W-SI vs. W-CTR group. Therefore, compared to Hu sheep, the testicular cells of Wugu–Hu sheep may be more sensitive to temperature increases or respond faster.

However, the difference in transcriptome was not reflected in sperm motility and defects, suggesting that Wugu–Hu rams may possess a potential compensatory mechanism within their testicular cells to mitigate the impact of heat stress on semen quality. The clustering patterns in Figure 5E,F provide insights into potential compensatory mechanisms underlying spermatogenesis recovery under heat stress. The downregulation of cell cycle-related genes in Wugu–Hu ram suggests inhibiting spermatogonia mitosis and spermatocyte meiosis during heat stress. This impact may result in DNA damage in germ cells, decreasing sperm PMN and motility [51,52]. However, the apoptosis pathway in upregulated gene clusters indicates a protective cellular response that may facilitate spermatogenic recovery [53,54]. It can be inferred that as indicated by the transcriptomic results, more germ cells in Wugu–Hu rams were affected by heat stress and sustained damage than in Hu rams. However, due to the apoptotic mechanisms, the damaged cells did not progress to form mature sperm. This is also consistent with the increase in the apoptosis rate in this study (Figure 8). As a result, differences in sperm motility and the PMN between the two sheep breeds were limited. The significant decline in sperm concentration of Wugu–Hu rams also reflects the higher numbers of apoptotic germ cells during spermatogenesis, which also provides evidence for this inference.

In the GO analysis of the W-SI vs. W-CTR group, based on BP screening, a total of 103 terms were significantly enriched (Table S1), including multiple terms directly related to the process of spermatogenesis, such as spermatogenesis, spermatid development, male meiosis I, male meiotic nuclear division, meiotic cell cycle. Highly relevant terms include cilium assembly, axoneme assembly, flagellated sperm motility, cilium dependent cell motility, binding of sperm to zona pellucida, acrosome reaction, and fertilization. This indicates that the expression of genes related to spermatogenesis undergoes significant changes after being subjected to thermal stimulation.

The top two pathways most relevant to spermatogenesis were selected from the significantly enriched KEGG pathways. PPI analysis was performed on significantly downregulated DEGs in the pathway, screening for the key genes CDK1, CDK2, CDC20, and PLK1. CDK1 is active in the late stage of meiosis, while CDK2 plays a key role in homologous recombination in the early stage of meiosis, jointly regulating the cell cycle. Reduced CDK1 and CDK2 expression may impair the initiation and progression of meiosis, potentially leading to defective homologous recombination and chromosomal missegregation [55,56]. CDC20 is a key regulatory factor for the spindle assembly checkpoint in the cell cycle [57]. PLK1 is a key kinase regulating meiosis and cell cycle in spermatogenesis [58]. PLK1 regulates the activity of CDC20 and CDK1 through phosphorylation, ensuring the timing and accuracy of meiosis [59]. CDC20 is a key activator of Anaphase Promoting Complex/Cyclosome (APC/C), and the activity of APC/C is regulated by CDK1 and PLK1, ensuring the accuracy of chromosome segregation [60,61]. In addition, the two key pathways we selected showed significant downregulation of a large number of DEGs. The downregulation of these genes could lead to impaired spermatogonia proliferation, defective meiotic progression, and increased apoptosis of developing germ cells [60,62]. This could ultimately result in reduced sperm production and compromised fertility. Although these more significant transcriptome changes compared to Hu sheep may suggest that Wugu–Hu sheep are more sensitive to temperature and have a smaller range of thermal neutral zones, the effective compensatory regulatory mechanisms in Wugu–Hu sheep minimized the impact of germ cell damage on sperm quality, which are worth further exploration in the future.

Genes related to meiosis were selected from the KEGG pathway for RT-qPCR validation experiments. They were all significantly downregulated in the Wugu–Hu sheep post-insulation group, indicating that the mild scrotal heat stress in the crossbreed sheep caused impaired spermatogenesis function. However, the same genes showed no significant differences in the Hu sheep group (Table S2).

Overall, although there were significant differences in gene expression changes in testicular cells between the two breeds of sheep under thermal stimulation in this experiment, this difference was not reflected in changes in semen quality, possibly due to multiple compensatory mechanisms at the cellular level. In addition to the compensatory mechanism of cell apoptosis demonstrated in this study, heat shock proteins may have played a crucial role in cellular protection by assisting protein refolding and minimizing cellular stress [63,64]. Another possibility is that post-transcriptional regulation (e.g., miRNA-mediated regulation) modulated gene expression at the protein level, preventing significant phenotypic disruptions despite transcriptomic changes [65]. However, since this study’s transcriptomic results include multiple testicular cell types, mainly including germ cells, Sertoli cells, and Leydig cells, many proteins are expressed across all cell types. Therefore, the transcriptomic data alone are insufficient to support detailed analyses of the roles of specific proteins in heat stress protection mechanisms. As demonstrated by the TUNEL results in this study, although apoptosis rates were significantly observed in specific cell types, apoptotic cells constituted only a small part of the total testicular cells. Consequently, the differential expression of key apoptosis-related genes (Bcl2, Bax) in the transcriptomic results was not significant at the whole-tissue level. Notably, meiosis occurs exclusively in germ cells within the testes, so the transcriptomic analysis’s primary focus was proteins related to the meiotic process.

Future research should employ multi-omics approaches, including transcriptomics, proteomics, metabolomics, and single-cell RNA sequencing, to discover cell-type-specific compensatory mechanisms during heat stress recovery. Detailed investigations into apoptosis, heat shock proteins, and post-transcriptional regulation explain phenotypic outcomes. Long-term studies, such as multi-seasonal breeding trials, assess the lasting impacts of heat stress on fertility and genetic stability.

## 5. Conclusions

The spermatogenesis-related genes in Wugu–Hu sheep were significantly downregulated after mild scrotal heat treatment, demonstrating that Wugu–Hu sheep are more sensitive to heat stress than Hu sheep. However, their sperm quality recovery followed a similar trend, demonstrating that compensatory mechanisms at the cellular level helped mitigate heat-induced damage. These findings show the complexity of heat stress adaptation in ram testicular cells and highlight the role of apoptosis in maintaining spermatogenesis. The results provide novel insights into the resilience of spermatogenesis under mild heat stress and contribute to an understanding of breed-specific molecular responses to heat stress.

## Data Availability

The datasets generated and analyzed during the current study are available from the corresponding author upon reasonable request.

## Supplementary Materials

The following supporting information can be downloaded at: https://www.mdpi.com/article/10.3390/ani15050724/s1, Figure S1: Scrotal insulation bag with temperature and humidity sensor; Figure S2: Scrotal surface humidity in 3 days; Table S1. GO analysis in BP ontology (W-SI vs W-CTR); Table S2. Gene primer sequences; Table S3A. Differential gene expression in W-SI vs W-CTR group; Table S3B. Differential gene expression in H-SI vs H-CTR group; Table S4 Significantly enriched pathways of DEGs associated with spermatogenesis; Table S5 Effect of Scrotal Insulation on Semen Quality, PMN, and Sperm Concentration in Wugu-Hu and Hu Sheep.

## Abbreviations

The following abbreviations are used in this manuscript:

AV	Artificial vagina
BP	Biological Process
CASA	Computer-aided sperm analysis
CC	Cellular Component
CTR	Control
CI	Confidence interval
DEG	Differentially expressed gene
DNA	Deoxyribonucleic acid
EDTA	Ethylenediaminetetraacetic acid
FC	Fold change
FSH	Follicle-stimulating hormone
GO	Gene Ontology
H-SI	Hu ram with scrotal insulation
KEGG	Kyoto Encyclopedia of Genes and Genomes
LH	Luteinizing hormone
MF	Molecular Function
mRNA	Messenger ribonucleic acid
NCBI	National Center for Biotechnology Information
PBS	Phosphate-buffered saline
PCR	Polymerase chain reaction
PMN	Percentage morphologically normal
PPI	Protein-protein interaction
qPCR	Quantitative polymerase chain reaction
RNA	Ribonucleic acid
ROS	Reactive oxygen species
SE	Standard error
SI	Scrotal insulation
THI	Temperature–Humidity Index
W-SI	Wugu–Hu ram with scrotal insulation
