# Supplementary material for: Semen Quality, Testicular Cell Apoptosis, and Transcriptome Analysis Following Mild Scrotal Heat Stress in Wugu–Hu Crossbred and Hu Rams
Source: Animals (Basel). 2025 Mar 3;15(5):724. doi: 10.3390/ani15050724 (PMC11898594; doi:10.3390/ani15050724)
Supplement: Supplementary file 1 [file animals-15-00724-s001.zip › animals-3477973-supplementary.pdf]

**Supplementary materials**

**Figure S1.** Scrotal insulation bag (Fig. S1A) with temperature and humidity sensor (Fig. S1B).

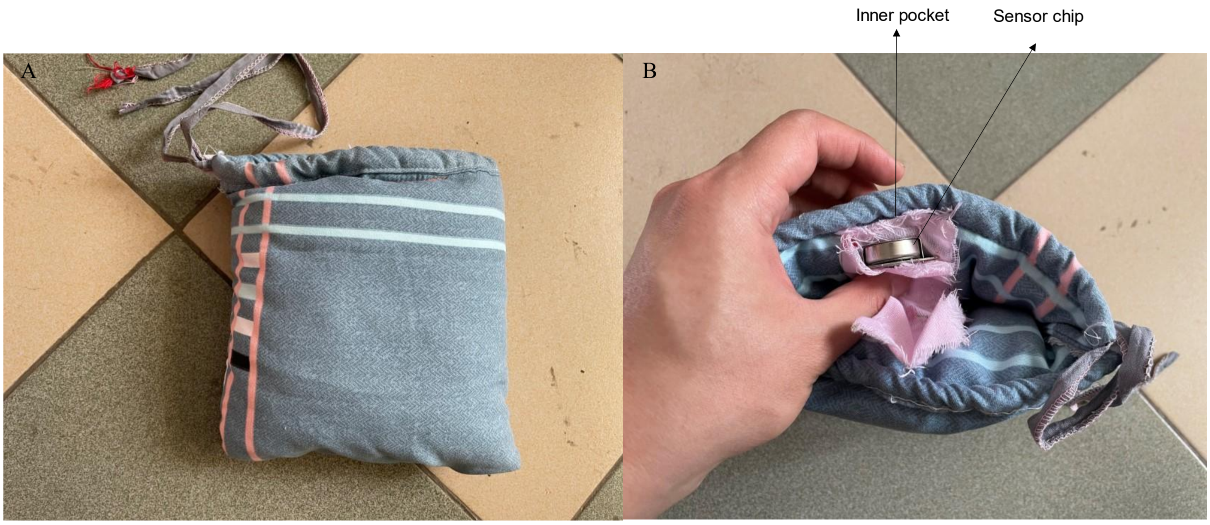


**Figure S2. Scrotal surface humidity in 3 days.**

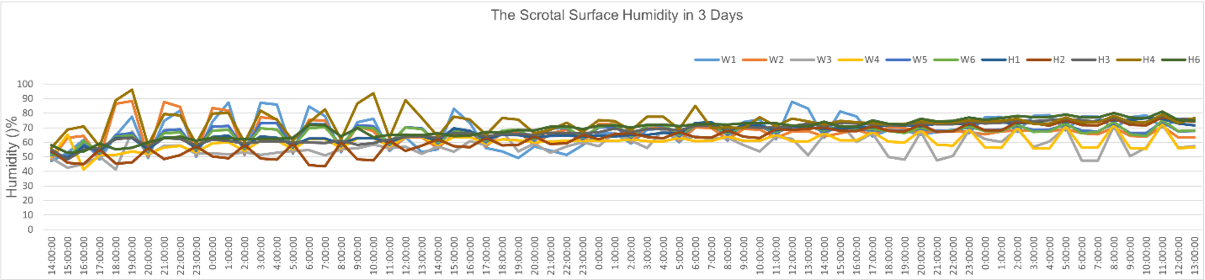

**Table S1. GO analysis in BP ontology (W-SI vs W-CTR).**

| ID | Description | pval | Count |
|---|---|---|---|
| GO:0007283 | Spermatogenesis | 1.91064E-23 | 248 |
| GO:0007155 | Cell adhesion | 3.47203E-18 | 256 |
| GO:0003341 | Cilium movement | 1.80895E-13 | 39 |
| GO:0016477 | Cell migration | 3.22131E-12 | 139 |
| GO:0030198 | Extracellular matrix organization | 4.27912E-12 | 122 |
| GO:0007275 | Multicellular organism development | 1.8517E-10 | 268 |
| GO:0030317 | Flagellated sperm motility | 6.77671E-10 | 58 |
| GO:0030154 | Cell differentiation | 1.10874E-09 | 328 |
| GO:0006508 | Proteolysis | 2.35995E-08 | 111 |
| GO:0007018 | Microtubule-based movement | 6.90458E-08 | 58 |
| GO:0007160 | Cell-matrix adhesion | 6.82362E-08 | 62 |
| GO:0007339 | Binding of sperm to zona pellucida | 6.58241E-08 | 42 |
| GO:0050808 | Synapse organization | 7.28368E-08 | 43 |
| GO:0060271 | Cilium assembly | 1.02245E-07 | 124 |
| GO:0030199 | Collagen fibril organization | 2.09571E-07 | 50 |
| GO:0007156 | Homophilic cell adhesion via plasma membrane adhesion molecules | 3.73165E-07 | 86 |
| GO:0060285 | Cilium-dependent cell motility | 4.32491E-07 | 17 |
| GO:0006836 | Neurotransmitter transport | 5.2612E-07 | 40 |
| GO:0007129 | Homologous chromosome pairing at meiosis | 8.76699E-07 | 26 |
| GO:0035082 | Axoneme assembly | 8.78672E-07 | 20 |
| GO:0001525 | Angiogenesis | 1.25527E-06 | 119 |
| GO:0003351 | Epithelial cilium movement involved in extracellular fluid movement | 2.06386E-06 | 13 |
| GO:0008360 | Regulation of cell shape | 2.50225E-06 | 80 |
| GO:0034446 | Substrate adhesion-dependent cell spreading | 3.85644E-06 | 40 |
| GO:0098609 | Cell-cell adhesion | 4.00395E-06 | 73 |
| GO:0007411 | Axon guidance | 5.51819E-06 | 111 |
| GO:0034220 | Ion transmembrane transport | 6.27105E-06 | 66 |
| GO:0007165 | Signal transduction | 9.3029E-06 | 328 |
| GO:0048167 | Regulation of synaptic plasticity | 9.74674E-06 | 30 |
| GO:0007507 | Heart development | 1.13326E-05 | 87 |
| GO:0006749 | Glutathione metabolic process | 1.15359E-05 | 33 |
| GO:0007613 | Memory | 1.24806E-05 | 40 |
| GO:0045165 | Cell fate commitment | 1.24368E-05 | 36 |
| GO:0051480 | Regulation of cytosolic calcium ion concentration | 1.25986E-05 | 25 |
| GO:0007420 | Brain development | 1.30999E-05 | 107 |
| GO:0036158 | Outer dynein arm assembly | 1.80191E-05 | 18 |
| GO:0035725 | Sodium ion transmembrane transport | 2.1084E-05 | 41 |
| GO:0002576 | Platelet degranulation | 2.15136E-05 | 32 |
| GO:0051965 | Positive regulation of synapse assembly | 2.203E-05 | 33 |

| | | | |
|---|---|---|---|
| GO:0045766 | Positive regulation of angiogenesis | 2.28639E-05 | 58 |
| GO:0046513 | Ceramide biosynthetic process | 2.87513E-05 | 25 |
| GO:0055085 | Transmembrane transport | 3.71062E-05 | 67 |
| GO:0034587 | Pirna processing | 3.9346E-05 | 14 |
| GO:0007169 | Transmembrane receptor protein tyrosine kinase signaling pathway | 3.99317E-05 | 66 |
| GO:0031274 | Positive regulation of pseudopodium assembly | 4.23781E-05 | 10 |
| GO:0060317 | Cardiac epithelial to mesenchymal transition | 4.23781E-05 | 10 |
| GO:0018146 | Keratan sulfate biosynthetic process | 4.8709E-05 | 12 |
| GO:0007605 | Sensory perception of sound | 4.93234E-05 | 72 |
| GO:0016525 | Negative regulation of angiogenesis | 5.03113E-05 | 44 |
| GO:0007099 | Centriole replication | 5.44021E-05 | 18 |
| GO:0007229 | Integrin-mediated signaling pathway | 6.46498E-05 | 54 |
| GO:0007286 | Spermatid development | 7.8726E-05 | 51 |
| GO:0034765 | Regulation of ion transmembrane transport | 7.81339E-05 | 66 |
| GO:0007413 | Axonal fasciculation | 9.47941E-05 | 13 |
| GO:0036159 | Inner dynein arm assembly | 9.47941E-05 | 13 |
| GO:0050982 | Detection of mechanical stimulus | 9.47941E-05 | 13 |
| GO:0071805 | Potassium ion transmembrane transport | 9.43531E-05 | 59 |
| GO:0008285 | Negative regulation of cell population proliferation | 0.000100314 | 158 |
| GO:0002040 | Sprouting angiogenesis | 0.000111195 | 23 |
| GO:0007141 | Male meiosis I | 0.00011767 | 17 |
| GO:0007219 | Notch signaling pathway | 0.000117472 | 56 |
| GO:0043588 | Skin development | 0.000118166 | 24 |
| GO:0009612 | Response to mechanical stimulus | 0.000123565 | 25 |
| GO:0016042 | Lipid catabolic process | 0.000123958 | 50 |
| GO:0030501 | Positive regulation of bone mineralization | 0.000123565 | 25 |
| GO:0070374 | Positive regulation of ERK1 and ERK2 cascade | 0.000135324 | 94 |
| GO:0007338 | Single fertilization | 0.000183406 | 44 |
| GO:0010951 | Negative regulation of endopeptidase activity | 0.000214925 | 32 |
| GO:0035556 | Intracellular signal transduction | 0.000223178 | 168 |
| GO:0009566 | Fertilization | 0.000231981 | 29 |
| GO:0051497 | Negative regulation of stress fiber assembly | 0.000235935 | 25 |
| GO:0070588 | Calcium ion transmembrane transport | 0.000291952 | 36 |
| GO:0007130 | Synaptonemal complex assembly | 0.00029693 | 17 |
| GO:0070831 | Basement membrane assembly | 0.000311389 | 10 |
| GO:0032488 | Cdc42 protein signal transduction | 0.000317659 | 8 |
| GO:0030036 | Actin cytoskeleton organization | 0.000331802 | 83 |
| GO:0050679 | Positive regulation of epithelial cell proliferation | 0.000353131 | 32 |
| GO:0006821 | Chloride transport | 0.00038189 | 30 |
| GO:0009888 | Tissue development | 0.000429232 | 23 |
| GO:0051017 | Actin filament bundle assembly | 0.000422436 | 22 |
| GO:0001764 | Neuron migration | 0.000443098 | 56 |
| GO:0001558 | Regulation of cell growth | 0.000535746 | 33 |

| | | | |
|---|---|---|---|
| GO:0007340 | Acrosome reaction | 0.000530912 | 15 |
| GO:0051321 | Meiotic cell cycle | 0.000534557 | 45 |
| GO:0014068 | Positive regulation of phosphatidylinositol 3-kinase signaling | 0.000564571 | 32 |
| GO:0007157 | Heterophilic cell-cell adhesion via plasma membrane cell adhesion molecules | 0.000593595 | 31 |
| GO:1902476 | Chloride transmembrane transport | 0.000593595 | 31 |
| GO:0006509 | Membrane protein ectodomain proteolysis | 0.000608599 | 16 |
| GO:2001238 | Positive regulation of extrinsic apoptotic signaling pathway | 0.000608599 | 16 |
| GO:0007517 | Muscle organ development | 0.000619389 | 43 |
| GO:0007266 | Rho protein signal transduction | 0.000650958 | 29 |
| GO:0007140 | Male meiotic nuclear division | 0.000670873 | 17 |
| GO:0010718 | Positive regulation of epithelial to mesenchymal transition | 0.000678471 | 28 |
| GO:0046777 | Protein autophosphorylation | 0.000675278 | 83 |
| GO:0005975 | Carbohydrate metabolic process | 0.000745776 | 67 |
| GO:0007626 | Locomotory behavior | 0.000732213 | 35 |
| GO:0019228 | Neuronal action potential | 0.000751623 | 19 |
| GO:0035855 | Megakaryocyte development | 0.000752715 | 12 |
| GO:0042073 | Intraciliary transport | 0.000751623 | 19 |
| GO:0003222 | Ventricular trabecula myocardium morphogenesis | 0.000778887 | 9 |
| GO:0060294 | Cilium movement involved in cell motility | 0.000778887 | 9 |
| GO:0006936 | Muscle contraction | 0.000827288 | 39 |
| GO:0097062 | Dendritic spine maintenance | 0.000869609 | 7 |
| GO:0005576 | Extracellular region | 3.79602E-18 | 704 |
| GO:0031012 | Extracellular matrix | 6.81415E-16 | 148 |
| GO:0009986 | Cell surface | 3.45828E-15 | 276 |
| GO:0031514 | Motile cilium | 2.67878E-14 | 80 |
| GO:0005930 | Axoneme | 4.26751E-14 | 84 |
| GO:0001669 | Acrosomal vesicle | 1.80123E-13 | 70 |
| GO:0062023 | Collagen-containing extracellular matrix | 2.45118E-13 | 135 |
| GO:0005737 | Cytoplasm | 3.28725E-11 | 2289 |
| GO:0036126 | Sperm flagellum | 5.50691E-11 | 57 |
| GO:0005929 | Cilium | 5.21728E-10 | 129 |
| GO:0016324 | Apical plasma membrane | 2.15207E-09 | 189 |
| GO:0005856 | Cytoskeleton | 6.31881E-09 | 230 |
| GO:0016323 | Basolateral plasma membrane | 8.34977E-09 | 121 |
| GO:0005814 | Centriole | 1.34331E-08 | 94 |
| GO:0005788 | Endoplasmic reticulum lumen | 2.24558E-08 | 106 |
| GO:0045121 | Membrane raft | 9.51612E-08 | 137 |
| GO:0036064 | Ciliary basal body | 1.10433E-07 | 93 |
| GO:0005912 | Adherens junction | 1.49897E-07 | 88 |
| GO:0005874 | Microtubule | 2.14419E-07 | 171 |
| GO:0030425 | Dendrite | 2.04588E-07 | 220 |
| GO:0005858 | Axonemal dynein complex | 2.75138E-07 | 15 |
| GO:0070062 | Extracellular exosome | 3.23383E-07 | 433 |

| | | | |
|---|---|---|---|
| GO:0005901 | Caveola | 6.37581E-07 | 44 |
| GO:0005925 | Focal adhesion | 7.77686E-07 | 158 |
| GO:0043025 | Neuronal cell body | 7.71519E-07 | 170 |
| GO:0045211 | Postsynaptic membrane | 7.54519E-07 | 81 |
| GO:0032809 | Neuronal cell body membrane | 1.48981E-06 | 21 |
| GO:0009897 | External side of plasma membrane | 2.50973E-06 | 161 |
| GO:0030424 | Axon | 2.53986E-06 | 158 |
| GO:0015629 | Actin cytoskeleton | 3.70398E-06 | 116 |
| GO:0030175 | Filopodium | 3.92303E-06 | 44 |
| GO:0097228 | Sperm principal piece | 4.99601E-06 | 18 |
| GO:0005615 | Extracellular space | 5.92325E-06 | 697 |
| GO:0001725 | Stress fiber | 7.03335E-06 | 41 |
| GO:0043235 | Receptor complex | 1.0272E-05 | 96 |
| GO:0036157 | Outer dynein arm | 1.54769E-05 | 11 |
| GO:0042383 | Sarcolemma | 1.71865E-05 | 56 |
| GO:0000795 | Synaptonemal complex | 1.80191E-05 | 18 |
| GO:0005813 | Centrosome | 2.19994E-05 | 242 |
| GO:0031410 | Cytoplasmic vesicle | 6.04039E-05 | 150 |
| GO:0005911 | Cell-cell junction | 6.54841E-05 | 87 |
| GO:0048786 | Presynaptic active zone | 7.27671E-05 | 32 |
| GO:0098978 | Glutamatergic synapse | 7.2672E-05 | 155 |
| GO:0008076 | Voltage-gated potassium channel complex | 8.30612E-05 | 43 |
| GO:0005604 | Basement membrane | 0.000101024 | 53 |
| GO:0031528 | Microvillus membrane | 0.00011767 | 17 |
| GO:0005794 | Golgi apparatus | 0.00013188 | 413 |
| GO:0030018 | Z disc | 0.000157331 | 69 |
| GO:0042734 | Presynaptic membrane | 0.00018412 | 36 |
| GO:0098688 | Parallel fiber to Purkinje cell synapse | 0.000200888 | 15 |
| GO:0097225 | Sperm midpiece | 0.000237765 | 26 |
| GO:0000775 | Chromosome, centromeric region | 0.00042752 | 48 |
| GO:0043197 | Dendritic spine | 0.000423096 | 77 |
| GO:0034707 | Chloride channel complex | 0.000622515 | 30 |
| GO:0031234 | Extrinsic component of cytoplasmic side of plasma membrane | 0.000643757 | 37 |
| GO:0005940 | Septin ring | 0.000752715 | 12 |
| GO:0032153 | Cell division site | 0.000752715 | 12 |
| GO:0098686 | Hippocampal mossy fiber to CA3 synapse | 0.000751623 | 19 |
| GO:0042824 | MHC class I peptide loading complex | 0.000869609 | 7 |
| GO:0005509 | Calcium ion binding | 2.43156E-28 | 413 |
| GO:0050839 | Cell adhesion molecule binding | 3.71827E-08 | 44 |
| GO:0004222 | Metalloendopeptidase activity | 4.26752E-07 | 77 |
| GO:0005524 | ATP binding | 4.74879E-07 | 736 |
| GO:0008201 | Heparin binding | 1.84217E-06 | 94 |
| GO:0008569 | Minus-end-directed microtubule motor activity | 2.69446E-06 | 17 |
| GO:0005516 | Calmodulin binding | 3.26192E-06 | 121 |

| | | | |
|---|---|---|---|
| GO:0005201 | Extracellular matrix structural constituent | 6.05503E-06 | 49 |
| GO:0005251 | Delayed rectifier potassium channel activity | 6.86472E-06 | 22 |
| GO:0005102 | Signaling receptor binding | 7.26756E-06 | 142 |
| GO:0050840 | Extracellular matrix binding | 1.12725E-05 | 20 |
| GO:0003777 | Microtubule motor activity | 1.2601E-05 | 38 |
| GO:0003779 | Actin binding | 1.27417E-05 | 135 |
| GO:0008017 | Microtubule binding | 1.57799E-05 | 138 |
| GO:0004252 | Serine-type endopeptidase activity | 1.60921E-05 | 94 |
| GO:0004714 | Transmembrane receptor protein tyrosine kinase activity | 1.83167E-05 | 67 |
| GO:1990837 | Sequence-specific double-stranded DNA binding | 4.53444E-05 | 201 |
| GO:0003700 | DNA-binding transcription factor activity | 5.64791E-05 | 198 |
| GO:0051015 | Actin filament binding | 5.93339E-05 | 113 |
| GO:0008237 | Metallopeptidase activity | 7.47062E-05 | 45 |
| GO:0015293 | Symporter activity | 0.00010058 | 39 |
| GO:0048407 | Platelet-derived growth factor binding | 0.000116029 | 9 |
| GO:0005543 | Phospholipid binding | 0.00014166 | 48 |
| GO:0005178 | Integrin binding | 0.000164238 | 79 |
| GO:0005249 | Voltage-gated potassium channel activity | 0.000192235 | 35 |
| GO:0005518 | Collagen binding | 0.000214925 | 32 |
| GO:0005080 | Protein kinase C binding | 0.000237519 | 27 |
| GO:0043394 | Proteoglycan binding | 0.000317659 | 8 |
| GO:0005262 | Calcium channel activity | 0.000322632 | 34 |
| GO:0004713 | Protein tyrosine kinase activity | 0.000377301 | 39 |
| GO:0002020 | Protease binding | 0.000401406 | 38 |
| GO:0001228 | DNA-binding transcription activator activity, RNA polymerase II-specific | 0.000423337 | 185 |
| GO:0004364 | Glutathione transferase activity | 0.000429232 | 23 |
| GO:0004435 | Phosphatidylinositol phospholipase C activity | 0.00043871 | 14 |
| GO:0030165 | PDZ domain binding | 0.000601457 | 48 |
| GO:0051959 | Dynein light intermediate chain binding | 0.000778673 | 23 |
| GO:0005021 | Vascular endothelial growth factor receptor activity | 0.000869609 | 7 |

Table S2. Gene primer sequences.

| Genes | Primers sequence (5’- >3’) | Accession number | Product length (bp) |
|---|---|---|---|
| SYCP2 | F: ACCACCACTGCAAATGACGA<br>R: CTGCTGCCTGTGAATCGAGA | XM_027976988 | 242 |
| SYCP3 | F: TCCGGGAAGTTGGCAAAACC<br>R: GGTCTTCTCTTCAATGGCATCC | XM_019961171.1 | 117 |
| CDK1 | F: ATGGCTTGGATCTGCTCTCG<br>R: TGCTCTTGACACAACACAGGA | NM_001142508.1 | 154 |
| DDX4 | F: AGTGCCCTGTTCTTGTTGCT<br>R: ACGACCAGTACGCCCAATTC | XM_054352837 | 131 |
| TNP1 | F: GGCATGA GGAGGGGCAAGAAC<br>R: TCACAAGTGGGAGCGCAAATTG | NM_003284 | 138 |
| β-Actin | F: CTCTTCCAGCCTTCCTTCCT<br>R: GGGCAGTGATCTCTTTCTGC | NM_001101 | 178 |

Table S3A. Differential gene expression in W-SI vs W-CTR group

| Genes | BaseMean | Log2FoldChange | padj | Up/Down | Significant |
|---|---|---|---|---|---|
| SYCP2 | 373.2611 | -3.31832 | 0.000771554 | down | yes |
| SYCP3 | 1181.179 | -3.1935 | 1.42E-16 | down | yes |
| CDK1 | 93.93165 | -3.202823606 | 1.50E-15 | down | yes |
| DDX4 | 2582.497 | -3.26615 | 3.68E-23 | down | yes |
| TNP1 | 1697.903 | -3.78439 | 2.55E-05 | down | yes |

Table S3B. Differential gene expression in H-SI vs H-CTR group

| Genes | BaseMean | Log2FoldChange | padj | Up/Down | Significant |
|---|---|---|---|---|---|
| SYCP2 | 2068.168 | 0.079455535 | 0.84680444 | up | no |
| SYCP3 | 267.0271 | -0.393669119 | 0.374167887 | down | no |
| CDK1 | 160.484196 | 0.139946713 | 0.787100424 | up | no |
| DDX4 | 5105.318 | 0.212797637 | 0.533122991 | up | no |
| TNP1 | 8805.094 | 0.884308519 | 0.122281352 | up | no |

Table S4 Significantly enriched pathways of DEGs associated with spermatogenesis.

| Map | Name | pval | Up_Gene | Down_Gene |
|---|---|---|---|---|
| map04114 | Oocyte meiosis | 2E-04 | ADCY4\|K08044;ADCY5\|K08045;ADCY8\|K08048;CALML4\|K02183;CAMK2A\|K04515;CAMK2G\|K04515;IGF1\|K05459;IGF1R\|K05087;ITPR2\|K04959;LOC114117278\|K06631;LOC114118477\|K05868;LOC121816477\|K08047;MAP2K1\|K04368;MAPK11\|K04441;MAPK12\|K04441;MAPK14\|K04441;PPP2CB\|K04382;PPP2R1A\|K03456;PPP2R5B\|K11584;PPP3CA\|K04348;PPP3R1\|K06268;PRKACB\|K04345;RPS6KA3\|K04373;YWHAB\|K16197;YWHAG\|K16198;YWHAH\|K16198;YWHAZ\|K16197 | ADCY1\|K08041;ANAPC10\|K03357;ANAPC11\|K03358;ANAPC2\|K03349;ANAPC5\|K03352;AURKA\|K11481;BUB1\|K02178;CALM3\|K02183;CCNB1\|K05868;CCNB2\|K21770;CCNE1\|K06626;CCNE2\|K06626;CDC20\|K03363;CDC25C\|K05867;CDK1\|K02087;CDK2\|K02206;CPEB1\|K02602;CPEB2\|K02602;CPEB3\|K02602;CUL1\|K03347;ESPL1\|K02365;FBXO43\|K10318;FBXO5\|K10292;ITPR3\|K04960;LOC101107408\|K08694;LOC101114750\|K03359;LOC101117953\|K06269;LOC101121823\|K16198;LOC105609549\|K03363;LOC105614854\|K02183;LOC114112929\|K02183;LOC114112940\|K03094;LOC114117819\|K05868;LOC121817856\|K13055;MAD2L1\|K02537;PGR\|K08556;PKMYT1\|K06633;PLCZ1\|K05861;PLK1\|K06631;PPP1CC\|K06269;PPP2R5C\|K11584;PPP3R2\|K06268;PTTG1\|K06635;REC8\|K13054;SGO1\|K11580;SMC1B\|K06636;SPDYA\|K08694;STAG3\|K13055 |
| map04110 | Cell cycle | 0.015 | CCND3\|K10152;CDKN1A\|K06625;CDKN1B\|K06624;CHEK2\|K06641;E2F2\|K09389;GADD45A\|K04402;GADD45B\|K04402;GADD45G\|K04402;LOC105609509\|K02603;LOC114117278\|K06631;LOC114118477\|K05868;MYC\|K04377;RAD21\|K06670;SMAD3\|K23605;STAG2\|K06671;TGFB2\|K13376;TP53\|K04451;YWHAB\|K16197;YWHAG\|K16198;YWHAH\|K16198;YWHAZ\|K16197 | ANAPC10\|K03357;ANAPC11\|K03358;ANAPC2\|K03349;ANAPC5\|K03352;BUB1\|K02178;BUB1B\|K06637;BUB3\|K02180;CCNA1\|K06627;CCNA2\|K06627;CCNB1\|K05868;CCNB2\|K21770;CCND1\|K04503;CCNE1\|K06626;CCNE2\|K06626;CDC14A\|K06639;CDC20\|K03363;CDC25A\|K06645;CDC25C\|K05867;CDC45\|K06628;CDC6\|K02213;CDK1\|K02087;CDK2\|K02206;CDKN2C\|K06622;CDKN2D\|K06623;CHEK1\|K02216;CUL1\|K03347;DBF4\|K06629;E2F1\|K17454;ESPL1\|K02365;LOC101114750\|K03359;LOC101121823\|K16198;LOC105609549\|K03363;LOC114112940\|K03094;LOC114113021\|K06621;LOC114117819\|K05868;MAD2L1\|K02537;MCM2\|K02540;MCM4\|K02212;MCM5\|K02209;MCM6\|K02542;PCNA\|K04802;PKMYT1\|K06633;PLK1\|K06631;PTTG1\|K06635;RBL1\|K04681;SFN\|K06644;SMC1B\|K06636;TFDP1\|K04683;TGFB3\|K13377;TTK\|K08866;WEE2\|K06632 |

Table S5 Effect of Scrotal Insulation on Semen Quality, PMN, and Sperm Concentration in Wugu-Hu and Hu Sheep

| Day | Concentration (Spz/ml) | Progressive motility (%) | PMN (%) | Concentration (Spz/ml) | Progressive Motility | PMN (%) |
|---|---|---|---|---|---|---|
| | Wugu-Hu sheep | | | Hu sheep | | |
| CTR | $4.03\pm0.10^{a**}$ | $82.00\pm0.90^{a}$ | $97.35\pm0.29^{a}$ | $4.77\pm0.09^{a}$ | $82.15\pm2.67^{a}$ | $97.54\pm0.07^{a}$ |
| Day 11 | $4.59\pm0.42^{a*}$ | $39.83\pm6.96^{c}$ | $94.14\pm0.29^{c*}$ | $5.77\pm0.15^{c}$ | $37.45\pm5.86^{c}$ | $95.93\pm0.51^{b}$ |
| Day 16 | $4.14\pm0.26^{a}$ | $56.45\pm2.04^{c}$ | $95.18\pm0.66^{c}$ | $4.68\pm0.37^{a}$ | $63.58\pm4.63^{b}$ | $95.95\pm0.50^{b}$ |
| Day 21 | $3.11\pm0.22^{c}$ | $77.87\pm3.92^{a}$ | $94.75\pm0.63^{c}$ | $3.61\pm0.44^{b}$ | $83.06\pm3.22^{a}$ | $94.98\pm0.42^{c}$ |
| Day 26 | $4.63\pm0.41^{a}$ | $73.40\pm5.94^{a}$ | $95.42\pm0.51^{c}$ | $5.02\pm0.34^{a}$ | $80.92\pm3.37^{a}$ | $96.30\pm0.62^{a}$ |
| Day 31 | $3.26\pm0.09^{c}$ | $84.08\pm2.69^{a}$ | $94.21\pm0.64^{c}$ | $3.49\pm0.08^{c}$ | $89.91\pm1.19^{b}$ | $95.81\pm0.64^{b}$ |
| Day 36 | $2.78\pm0.22^{c}$ | $85.95\pm2.68^{a}$ | $93.29\pm0.56^{c}$ | $3.63\pm0.24^{c}$ | $87.20\pm2.10^{a}$ | $94.22\pm0.52^{c}$ |
| Day 41 | $3.13\pm0.39^{b}$ | $75.76\pm3.12^{a*}$ | $93.80\pm0.36^{c}$ | $4.18\pm0.18^{b}$ | $88.33\pm2.03^{a}$ | $94.19\pm0.52^{c}$ |
| Day 46 | $4.28\pm0.53^{a}$ | $84.58\pm2.57^{a*}$ | $94.50\pm0.61^{c}$ | $5.06\pm0.19^{a}$ | $90.32\pm0.76^{b}$ | $94.82\pm0.54^{c}$ |
| Day 51 | $3.83\pm0.18^{a**}$ | $83.70\pm1.55^{a}$ | $96.62\pm0.60^{a}$ | $4.68\pm0.11^{a}$ | $84.84\pm0.59^{a}$ | $96.16\pm0.66^{a}$ |
| Day 56 | $4.10\pm0.07^{a**}$ | $82.56\pm0.86^{a}$ | $97.26\pm0.45^{a}$ | $4.78\pm0.09^{a}$ | $83.63\pm1.31^{a}$ | $97.22\pm0.20^{a}$ |

*a, b, c: Differing subscripts denote a significant difference within the group compared to CTR, with a: no significant difference (P>0.05); b: significant difference (P<0.05); and c: highly significant difference (P<0.01). * Significant difference between breeds on the same day (P<0.05); ** highly significant difference (P<0.01). The lack of differing superscripts indicates p>0.05.*
